# Evaluation of the Remodeling of the Tricuspid Annulus and Right Heart in Persistent Atrial Fibrillation Patients With or Without Radiofrequency Ablation via Three‐Dimensional Echocardiography

**DOI:** 10.1111/echo.70319

**Published:** 2025-10-22

**Authors:** Yuan Tian, Yutian Wang, Bo Jing, Xiaofang Chen, Kunhui Huang, Jiancheng Xiu, Maolong Su, Qiuxia Zhang, Xu Chen

**Affiliations:** ^1^ Department of Echocardiography Xiamen Cardiovascular Hospital of Xiamen University Xiamen China; ^2^ Fujian Provincial Key Specialty Ultrasound Laboratory Fujian China; ^3^ Department of Cardiology Nanfang Hospital Southern Medical University Guangzhou Guangdong China; ^4^ Department of Cardiology Xiamen Cardiovascular Hospital of Xiamen University Xiamen China

**Keywords:** atrial fibrillation, radiofrequency ablation, right heart, three‐dimensional echocardiography, tricuspid annulus

## Abstract

**Background:**

Atrial fibrillation (AF) is linked to tricuspid annulus (TA) and right heart (RH) remodeling. Despite advances in catheter ablation, data regarding its structural impact on the TA and RH chambers remain limited. Therefore, the impact of radiofrequency ablation (RFA) on TA and RH remodeling in persistent AF patients is less clear. Furthermore, the potential of three‐dimensional echocardiography (3DE) for evaluating TA and RH remodeling following RFA requires further exploration. This study aimed to characterize TA and RH geometry and function in persistent AF patients before and after RFA via 3DE.

**Methods:**

The 90 subjects included in this study were divided into three groups: the persistent AF group (*n* = 30); persistent AF patients who underwent successful RFA with sinus rhythm maintenance at the 3‐month follow‐up were assigned to the RFA group (*n* = 30); and the control group (*n* = 30). 3DE datasets were analyzed to measure TA and RH geometry and function via Tomtec 3D echocardiography analysis software.

**Results:**

Compared with the AF group, the RFA group presented shorter linear dimensions of the right atrium (RA) (*p* < 0.05). The right atrial ejection fraction (EF) and right ventricular (RV) EF were significantly greater in the RFA group than in the AF group (RAEF: 45.56% vs. 28.57%, *p* = 0.003; RVEF: 45.94% vs. 38.45%, *p* < 0.001). However, there was no significant difference in the RV fractional area change (FAC) index (*p* > 0.05). The TA area, anteroposterior diameter, and circumference were significantly smaller in the RFA group than in the control group in all phases (all *p* < 0.05). After 3 months, RFA intervention (B = −0.440, *p* < 0.001) demonstrated significant inverse associations with the TA perimeter. Moreover, the non‐planar angle and tricuspid leaflet tenting height were smaller in the RFA group than in the control group in the systolic phase (*p* < 0.05).

**Conclusions:**

This study suggests that RFA may contribute to favorable TA and RH remodeling in patients with persistent AF, and that 3DE may provide more comprehensive and sensitive assessments with excellent feasibility, facilitating readily accessible evaluations for AF patients undergoing RFA.

## Graphical abstract

Three‐dimensional echocardiography (3DE) for evaluating the reverse remodeling of tricuspid annulus (TA) and right heart (RH) in persistent atrial fibrillation (AF) with radiofrequency ablation (RFA).

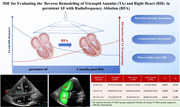

**Abbreviations**
2DE: two‐dimensional echocardiography
3DE: three‐dimensional echocardiography
AF: atrial fibrillation
ALT: alanine aminotransferase
ANOVA: analysis of variance
AP: anteroposterior
ARB: angiotensin II receptor blocker
ARNI: angiotensin receptor‐neprilysin inhibitor
AST: aspartate aminotransferase
BB: beta‐blocker
BMI: body mass index
BSA: body surface area
CMR: cardiac magnetic resonance
CT: computed tomography
DBP: diastolic blood pressure
DM: diabetes mellitus
ED: end‐diastolic
EDV: end‐diastolic volume
EF: ejection fraction
EROA: effective regurgitant orifice area
ES: end‐systolic
ESC: European Society of Cardiology
ESV: end‐systolic volume
FAC: fractional area change
LVEF: left ventricular ejection fraction
MDCT: multi‐detector row computed tomography
PISA: proximal isovelocity surface area
RA: right atrium
RAV: right atrial volume
RAVmax: maximum right atrial volume
RAVmin: minimum right atrial volume
RFA: radiofrequency ablation
RH: right heart
RV: right ventricle
RVEDV: right ventricular end‐diastolic volume
RVEF: right ventricular ejection fraction
RVESV: right ventricular end‐systolic volume
SBP: systolic blood pressure
SGLT2i: sodium‐glucose cotransporter 2 inhibitor
SL: septal–lateral
Src: serum creatinine
TA: tricuspid annulus
TL: tricuspid leaflet
TR: tricuspid regurgitation
TV: tricuspid valve
VCW: vena contracta width
VIF: variance inflation factor

## 1 Introduction

Atrial fibrillation (AF) is the most prevalent cardiac arrhythmia worldwide and contributes substantially to morbidity and mortality. Its incidence increases markedly with advancing age, and is particularly prevalent in patients with hypertension (HTN), heart failure, valvular disease, or structural heart disease [1]. Previous studies investigating the association between AF and tricuspid regurgitation (TR) have demonstrated that AF‐related TR represents atrial functional TR caused by leaflet malcoaptation secondary to right atrial (RA) dilation and tricuspid annular (TA) enlargement, with annular dilatation serving as the determinant of functional TR [2]. Notably, remodeling of the TA and RA has been observed even in AF patients without severe TR [3].

Previous studies have demonstrated that restoring sinus rhythm following radiofrequency ablation (RFA) for AF may reduce TR severity and even prevent right heart (RH) remodeling in AF patients with moderate or greater TR [4, 5, 6, 7]. Tomofumi et al. employed multi‐detector row computed tomography (MDCT) to evaluate TA changes before and after RFA, revealing that rhythm control facilitates reverse remodeling of the TA and reduces the right atrial volume (RAV) [8]. Furthermore, investigations utilizing computed tomography (CT) to analyze the anatomical characteristics associated with AF recurrence after RFA in paroxysmal and persistent AF patients revealed that post‐procedural RAV expansion and tricuspid valve (TV) diameter enlargement may indicate persistent electrical remodeling or underlying structural abnormalities, which are potentially related to high recurrence risk [9]. However, CT entails radiation exposure and does not provide real‐time functional assessment.

In contrast to CT, echocardiography serves as the preferred noninvasive cardiac imaging modality for evaluating TV structure and function because of its accessibility, rapid implementation, and absence of radiation exposure. Given that the TA has a complex three‐dimensional saddle‐shaped geometry rather than a simple planar structure [10, 11], conventional two‐dimensional echocardiography (2DE) remains insufficient for the comprehensive assessment of morphological characteristics and dynamic changes. Three‐dimensional echocardiography (3DE) enables dynamic visualization of TA motion patterns throughout the cardiac cycle [12]. Nevertheless, evidence regarding the detailed structural impact of RFA on TA and RH remodeling, particularly in persistent AF patients with or without TR, remains limited. Therefore, this study aimed to characterize the geometric structure and functional changes of the TA and RH following RFA in patients with persistent AF, with or without TR, using three‐dimensional echocardiography (3DE) with Tomtec Imaging Systems analysis software.

## 2 Methods

### 2.1 Study Design and Participants

**FIGURE 1:**
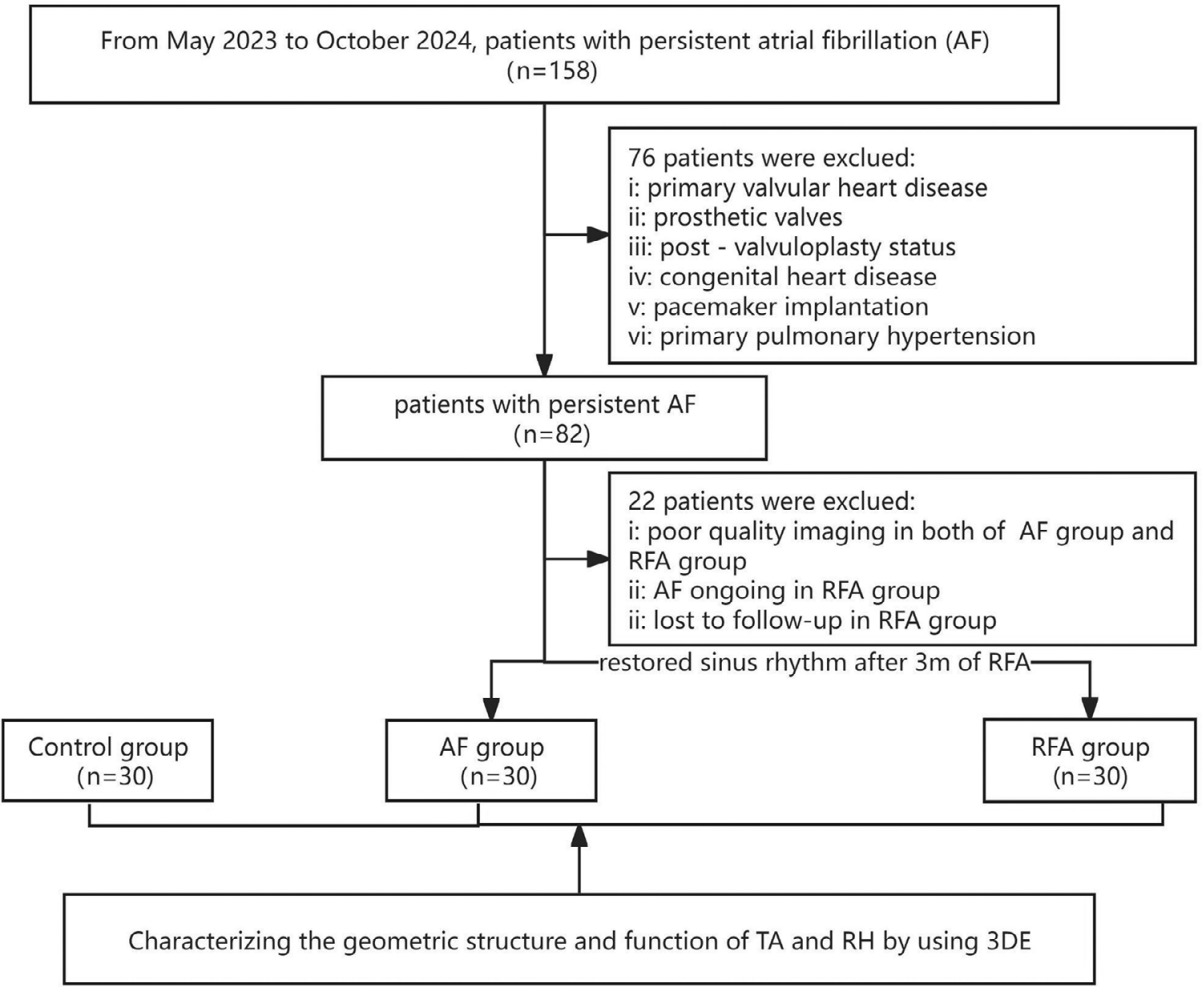
Flow chart of this study. 3DE, three‐dimensional echocardiography; AF, atrial fibrillation; RFA, radiofrequency ablation; RH, right heart; TA, tricuspid annulus.

From May 2023 to October 2024, 158 patients with persistent AF were recruited at Xiamen Cardiovascular Hospital Xiamen University. Those with left‐sided heart failure, primary valvular heart disease, prosthetic valves, post‐valvuloplasty status, congenital heart disease, cardiomyopathy, primary pulmonary hypertension, or pacemaker implantation were excluded. Eighty‐two patients were initially selected and divided into two groups. For patients in the AF group (*n* = 30), only baseline information was recorded. In contrast, patients who received RFA were followed up at 3 months (94.3 ± 3.1) post‐procedure. Among the patients, only those whose sinus rhythm was restored at follow‐up were included in the RFA group (*n* = 30). Additionally, 30 AF‐free individuals with high‐quality imaging data admitted during the same period served as the control group. Figure 1 shows the flow chart of this study.

AF diagnosis relies on electrocardiograms (ECGs) and medical records. The diagnostic criteria for AF and AF‐free status were based on the *2023 ACC/AHA/ACCP/HRS Guideline for the Diagnosis and Management of Atrial Fibrillation* [13]. According to the guideline, electrocardiographic characteristics of AF include: (1) irregular R‐R intervals (when atrioventricular conduction is present), (2) absence of distinct P waves, and (3) irregular atrial activity also known as fibrillatory waves. AF can be documented by, for example, 12‐lead ECG, rhythm strips, wearables, intracardiac electrograms, but will always require visual confirmation that the diagnosis is accurate. In our cohort, all patients underwent both 12‐lead ECG and 24‐h Holter monitoring to confirm persistent AF, and AF‐free status was determined by the absence of these findings during the same diagnostic procedures. The study was approved by the Ethics Committee of Xiamen Cardiovascular Hospital (No. XXY‐AF/SC‐09.01/2.0), and all participants provided written informed consent.

### 2.2 Echocardiographic Acquisition and Analysis

#### 2.2.1 Transthoracic Echocardiography Acquisition

A Philips EPIC 7C ultrasound diagnostic instrument equipped with a three‐dimensional cardiac probe X5‐1 (probe frequency of 1.3–4.8 MHz) was used.

First, the height, weight, systolic blood pressure (SBP), and diastolic blood pressure (DBP) of the subjects were measured and recorded. Body mass index (BMI) and body surface area (BSA) were calculated on the basis of height and weight. Second, comprehensive transthoracic echocardiography (TTE) was performed according to established guidelines. The participants were positioned in the left lateral decubitus position with simultaneous electrocardiographic monitoring, and five consecutive cardiac cycles were captured for analysis. Finally, standard apical four‐chamber views were acquired via 2DE.

Parameters were obtained for the RA transverse diameter, RV transverse diameter, tricuspid annulus (TA) diameter, mitral‐tricuspid annular angle, right ventricular fractional area change (RV FAC), left ventricular ejection fraction (LVEF; determined by Simpson's biplane method), and tricuspid annular plane systolic excursion (TAPSE) via M‐mode. Additionally, the vena contracta width (VCW) of the TR was measured via color Doppler, and the effective regurgitant orifice area (EROA) was derived from the proximal isovelocity surface area (PISA) methodology. The degrees of TR were classified based on 2023 ESC criteria: normal, mild TR(1+): VCW < 3 mm, EROA < 20 mm^2^; moderate TR(2+): VCW 3–6.9 mm, EROA 20–39 mm^2^; severe TR(3+): VCW 7–13.9 mm, EROA 40–59 mm^2^; massive TR(4+): VCW 14–20.9 mm, EROA 60–79 mm^2^; and torrential TR(5+): VCW ≥ 21 mm, EROA ≥ 80 mm^2^.

### 2.3 Three‐Dimensional Right Heart and Tricuspid Valve Quantitative Analysis

The Tomtec workstation 4D Assessment analysis software was used for offline analysis (Tomtec‐arena TTA2.31.04). Three‐dimensional (3D) images were acquired at the apex, ensuring that the tricuspid valve was clearly displayed at the center of the screen, with the complete outlines of the right atrium and right ventricle and the endocardium being clearly visible. The 3D full‐volume mode was activated, and the image depth was adjusted to a range of 12–16 cm while maintaining a frame rate of not less than one‐sixth of the heart rate. The direction of the probe sound beam was adjusted to be as perpendicular as possible to the TA, ensuring that the entire annulus was encompassed. The subject was instructed to hold their breath, following which image acquisition was initiated.

**FIGURE 2:**
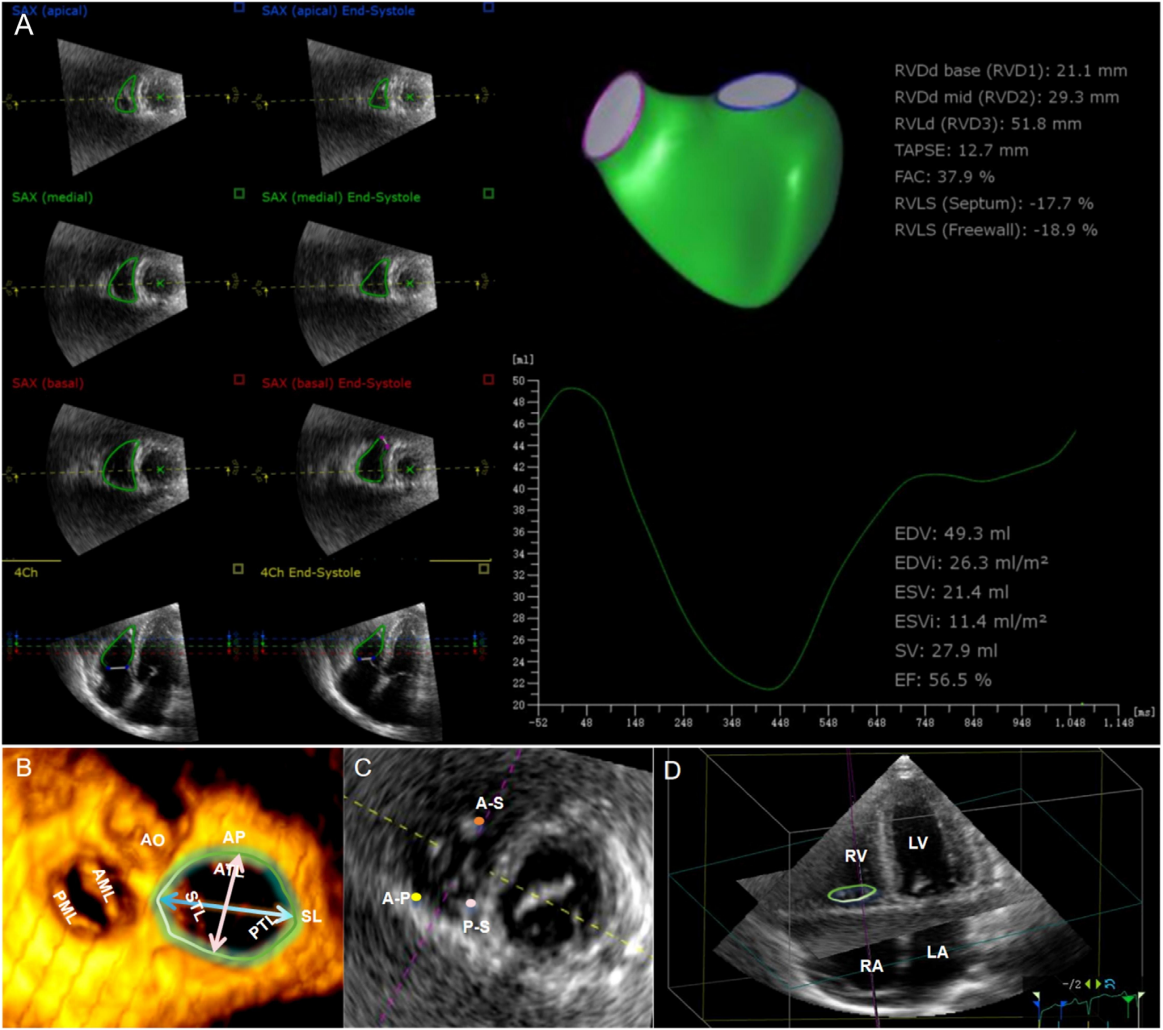
Measurement of TA and RH geometry and function via 3DE and Tomtec workstation 4D assessment analysis software. (A) Quantitative analysis of RV geometry and function via 4D assessment analysis software. The ventricular endocardium was detected at end‐diastole and end‐systole and was verified by the reader via long‐ and short‐axis views to obtain RV 3D reconstructions. The time‒volume curve of the right ventricle is automatically generated by the software, as are the chamber volumes and function. EDVi, end‐diastolic volume indexed; EF, ejection fraction; ESV, end‐systolic volume; ESVi, end‐systolic volume indexed; EVD, end‐diastolic volume; SV, stroke volume. (B, C) Enface ventricular view of the TA. In this case, the green circle represents the TA, the pink line represents the AP diameter, and the blue line represents the SL diameter. AML, anterior mitral leaflet; AO, aorta; AP, anteroposterior diameter; A‐P, anteroposterior commissure; ATL, anterior tricuspid leaflet; PML, posterior mitral leaflet; PTL, posterior tricuspid leaflet; STL, septal tricuspid leaflet; SL, septal–lateral diameter; A‐S, anteroseptal commissure; P‐S, posteroseptal commissure. (D) 3D reconstruction of the heart chambers. In this case, the green circle represents the TA. LA, left atrium; LV, left ventricle; RA, right atrium; RV, right ventricle.

Upon completion of 3D image acquisition and storage, the acquired 3D DICOM images were imported into the Tomtec workstation for offline analysis. The 4D assessment measurement software was launched. To optimize the visualization of the tricuspid valve, non‐relevant parts were removed. The multi‐planar reconstruction function was utilized to locate the region of interest precisely. The last frame prior to the commencement of tricuspid valve closure was defined as the RV end‐diastolic (ED) phase. At this point, the diameters of the TA, including the anteroposterior (AP) diameter, septal–lateral (SL) diameter, annulus circumference, annulus area, non‐planar angle, angle between the mitral and TA, and RV end‐diastolic volume (RV EDV), was measured. The sphericity index of the annulus (sphericity index = AP/SL) was calculated. The last frame before the tricuspid valve opened was defined as the RV end‐systolic (ES) phase. At this time, the systolic area of the TA and the RV end‐systolic volume (RV ESV) were measured, and the RV ejection fraction (RV EF) was calculated as follows: [(RV EDV‐RV ESV)/RV EDV]*100. All measurements were normalized for the BSA (Figure 2).

### 2.4 Statistical Analysis

Based on previous studies [3], the difference in the BSA‐adjusted tricuspid annular area between the AF group and the sinus rhythm group was approximately 1.4 cm^2^/m^2^. With a two‐sided *α* of 0.05, 90% power (1−*β*), and a 20% dropout rate, a minimum of 22 participants per group were estimated. Thus, this study included at least 22 AF patients, 22 patients with restored sinus rhythm post‐RFA, and 22 controls enrolled during the same period.

Categorical variables are expressed as numbers (*n*) and percentages (%). For continuous variables, normality was assessed via the Shapiro‒Wilk test, and homogeneity of variance was evaluated via Levene's test. Data conforming to a normal distribution are expressed as the means ± standard deviations (means ± SDs), whereas non‐normally distributed data are presented as medians and interquartile ranges (IQRs; first quartile‒third quartile).

For comparisons of categorical variables, the Chi‐square test was used. For continuous variables, if the data met the assumptions of normality and homogeneity of variance, one‐way analysis of variance (ANOVA) was applied for group comparisons. If the assumptions were not met, the non‐parametric Kruskal‒Wallis *H* test was used to assess overall significance. If the Kruskal‒Wallis *H* test indicated significant differences between groups, post hoc pairwise comparisons were conducted via the Mann‒Whitney *U* test, with *p* values adjusted via the Bonferroni correction to account for multiple comparisons.

In addition, sensitivity analyses were performed using multiple linear regression models to evaluate the robustness of the association between RFA and TA reverse remodeling (Additional file 1). Model 1 was adjusted for gender, age, and body surface area. Model 2 was further adjusted for systolic blood pressure, diastolic blood pressure, hypertension, diabetes mellitus, heart failure, stroke, smoking, and tricuspid regurgitation severity. Model 3 additionally adjusted for medications (angiotensin II receptor blocker, angiotensin receptor–neprilysin inhibitor, beta‐blocker, sodium–glucose cotransporter 2 inhibitor) and TA area at end‐diastole.

The threshold for statistical significance was set at *p* < 0.05. All the statistical analyses were performed via SPSS version 25.0 (IBM, USA) and R version 4.0.4.

## 3 Results

### 3.1 Baseline Characteristics

A total of 90 participants (54% male) were included in the final analysis to compare structural and functional remodeling across the three groups, comprising 30 AF patients who underwent RFA and 30 AF patients without RFA intervention, and 30 subjects without AF were included in the control group. The patients’ demographic characteristics are listed in Table 1. There were no statistically significant differences among the three groups (the mean age of the AF group was 64.03 ± 10.19 years, the mean age of the RFA group was 61.87 ± 9.51 years, and the mean age of the control group was 62.60 ± 8.46 years, *p* = 0.664). No statistically significant intergroup differences were observed in LVEF, NT‐pro BNP levels, hepatic function, or BSA measurements. No significant intergroup differences were observed in the incidence of hypertension, diabetes mellitus, or heart failure among the three groups (all *p* > 0.05). With respect to medication treatment, no significant intergroup disparities were noted in the use of angiotensin receptor blockers (ARBs), angiotensin receptor‐neprilysin inhibitors (ARNIs), beta‐blockers, or sodium‒glucose cotransporter‐2 inhibitors (SGLT2i) (all *p* > 0.05). Patients with AF presented a greater prevalence of TR than did the control group (*p* < 0.001). Three‐month post‐RFA follow‐up revealed significant clinical improvements in TR, especially among those with baseline severe TR (grade 3+). Furthermore, RFA‐treated patients had significantly lower serum creatinine concentrations than did the AF group (68.15 vs. 81.8 µmol/L, *p* = 0.027).

**TABLE 1:** **Baseline characteristics**.

	AF group	RFA group	control group	*p* ^a^	*p* ^b^	*p* value
	(*n* = 30)	(*n* = 30)	(*n* = 30)			
Age, years	64.03 ± 10.19	61.87 ± 9.51	62.60 ± 8.46	0.782	0.985	0.664
Gender, *n* (%)				0.795	1.000	0.956
Female	13(43.33)	14(46.67)	14(46.67)			
Male	17(56.67)	16(53.33)	16(53.33)			
Height (m)	1.66 ± 0.10	1.66 ± 0.10	1.63 ± 0.09	1.000	0.941	0.483
Weight (kg)	66.33 ± 13.3	66.53 ± 13.42	63.63 ± 11.65	1.000	1.000	0.621
BMI (kg/m^2^)	23.89 ± 3.39	24.00 ± 3.33	23.71 ± 3.03	1.000	1.000	0.941
BSA (m^2^)	1.74 ± 0.21	1.73 ± 0.21	1.69 ± 0.19	1.000	1.000	0.578
Heart rate (bpm)	82.20 ± 21.09	76.40 ± 10.06	72.97 ± 11.02	0.455	0.865	0.168
SBP (mm Hg)	129.67 ± 13.74	124.83 ± 14.58	127.43 ± 14.25	0.572	1.000	0.422
DBP (mm Hg)	79.27 ± 8.35	74.47 ± 7.79	74.73 ± 8.31	0.075	1.000	0.042
Hypertension, *n* (%)	17(56.67)	60(60)	17(56.67)	0.793	0.793	0.955
Diabetes, *n* (%)	5(16.67)	20(20)	2(6.67)	0.739	0.254	0.413
Heart Failure, *n* (%)	5(16.67)	30(30)	7(23.33)	0.222	0.559	0.475
Stroke, *n* (%)	6(20)	10(10)	1(3.33)	0.278	0.301	0.146
Smoking, *n* (%)	3(10)	20(20)	9(30)	0.472	0.371	0.153
NT‐pro BNP	462(107‐891)	104(97.5‐525.5)	165.5(83.88‐809.5)	0.288	1.000	0.247
ALT	19.5(13.05‐26.65)	15.3(12.95‐30.68)	17.9(13.13‐23.25)	1.000	1.000	0.856
AST	20.9(18.05‐27.5)	20.15(18.48‐30.9)	24.1(20‐26.23)	1.000	1.000	0.760
Albumin	42.1(39.25‐43.95)	40.2(39‐41.63)	41.25(40.15‐43.7)	0.276	0.12	0.096
Src	81.8(69.85‐85.9)	68.15(61.63‐86.03)	75(67.08‐89.88)	0.027	0.429	0.043
Albuminuria (−), *n* (%)	27(90)	93.33(93.33)	29(96.67)	1.000	0.492	0.514
LVEF (%)	64(58‐67)	61.35(57.75‐65.25)	64(59‐68.25)	1.000	0.342	0.293
TR degrees, *n* (%)				0.103	0.004	<0.001
0	7(23.33)	8(26.67)	21(70)			
1+	11(36.67)	16(53.33)	6(20)			
2+	7(23.33)	6(20)	3(10)			
3+	5(16.67)	0(0)	0(0)			
PASP	32.39 ± 6.05	29.79 ± 4.15	*29.89 ± 9.02	0.052		
ARB/ARNI, *n* (%)	11(36.7)	6(20)	8(26.7)	0.252	0.761	0.390
BB, *n* (%)	13(43.33)	50(50)	11(36.67)	0.605	0.297	0.581
SGLT2i, *n* (%)	7(23.33)	13.33(13.33)	6(20)	0.317	0.488	0.602

*Notes*: Data are presented as mean ± standard deviation, median (Q1–Q3) or percentage. *p* ^a^: RFA group compared with the AF group. *p* ^b^: RFA group compared with the control group.

Abbreviations: AF, atrial fibrillation; ALT, alanine aminotransferase; ARB, angiotensin II receptor blocker; ARNI, angiotensin receptor‐neprilysin inhibitor; AST, aspartate aminotransferase; BB, beta‐blocker; BMI, body mass index; BSA, body surface area; DBP, diastolic blood pressure; LVEF, left ventricular ejection fraction; PASP, pulmonary artery systolic pressure; RFA, radiofrequency ablation; SBP, systolic blood pressure; SGLT2i, sodium‐glucose cotransporter 2 inhibitor; Src, serum creatinine; TR, tricuspid regurgitation.

*For the control group, PASP was not measured in patients without tricuspid regurgitation; the values reported in the table represent the mean PASP of the nine patients with detectable tricuspid regurgitation.

### 3.2 Right Heart Chamber Geometry and Function

The echocardiographic characteristics of the RH chamber geometry and function are shown in Table 2. Compared with the AF group, the RFA group presented shorter linear dimensions, which included the maximum and minimum diameters of the RA (including both maximum and minimum RA diameters) (all *p* < 0.05). As expected, the RAEF and RVEF of the RFA group were significantly better than those of the AF group (RAEF: 45.56% vs. 28.57%, *p* = 0.003; RVEF: 45.94% vs. 38.45%, *p* < 0.001). However, there was no significant difference in the RV FAC index and the RV volume in both end‐diastole and end‐systole phases (*p* > 0.05).

**TABLE 2:** Parameters used for evaluating the geometry and function of right heart.

	AF group (*n* = 30)	RFA group (*n* = 30)	control group (*n* = 30)	*p* ^a^	*p* ^b^	*p* value
**Right atrial parameters measured by 3DE**						
RAVmax (mL)	65.6(52.7–98)	54.75(46.28–66.98)	36.95(29.73–48.6)	0.048	<0.001	<0.001
RAVmaxi (mL/m^2^)	39.65(30.91–53.05)	32.13(25.49–37.61)	22.54(18.11–28.27)	0.007	<0.001	<0.001
RAVmin (mL)	52.1(33.07–79.8)	28.6(20.02–41.57)	16.7(11.93–23.75)	<0.001	<0.001	<0.001
RAVmini (mL/m^2^)	28.14(20.24–39.32)	16.56(11.98–25.02)	9.54(7.31–13.9)	<0.001	<0.001	<0.001
RA EF (%)	28.57(22.18–34.85)	45.56(34.75–55.58)	52.79(45–58.86)	0.003	0.072	<0.001
**Right ventricular parameters measured by 3DE**						
RV EDV (mL)	103.4(84–126.85)	94.95(83.23–121.75)	76.75(69.63–86.3)	1.000	0.006	0.001
RV EDVi (mL/m^2^)	57.44(51.41–73.71)	59.84(48.49–69.75)	47.61(39.53–54.89)	1.000	0.016	0.002
RV ESV (mL)	69.5(43.6–79.7)	55.95(44.55–65.73)	39.15(33.6–47.88)	0.102	<0.001	<0.001
RV ESVi (mL/m^2^)	39.95(28.17–46.34)	31.88(26.62–35.89)	24.55(19.51–28.18)	0.090	0.003	<0.001
RV EF (%)	38.45(28.16–43.13)	45.94(42.34–49.34)	49.69(43.82–53.45)	<0.001	0.138	<0.001
**Right ventricular function measured by 2DE**						
RV FAC (%)	33.2(22.05–35.75)	35.4(32.03–41.13)	40.85(34.15–45.05)	0.063	0.138	0.001

*Notes*: Data are presented as mean ± standard deviation, median (Q1–Q3) or percentage. *p* ^a^: RFA group compared with the AF group. *p* ^b^: RFA group compared with the control group.

Abbreviations: 2DE, two dimension echocardiography; 3DE, three dimension echocardiography; RAVmax, the maximum diameter of right atrial volume; RAVmin, the minimum diameter of right atrial volume; RV EDV, the right – ventricular end – diastolic volume; RV ESV, the right – ventricular end – systolic volume; RV EF, the right – ventricular ejection fraction; RV FAC, the Right ventricular Fractional Area change.

Compared with the control group, the RFA group was still associated with longer RA linear dimensions and a larger RV volume (all *p* < 0.05). However, there were no significant differences in the RAEF, RVEF, or RV FAC (*p* > 0.05).

### 3.3 Tricuspid Annular Structure and Function

The tricuspid annular parameters derived from 3D echocardiography analysis are listed in Table 3. Compared with the AF group, the RFA group presented a significantly smaller index area, a smaller anteroposterior (AP) diameter and a smaller total circumference in the end‐diastole, middle‐systole, and end‐systole phases (all *p* < 0.05). The SL diameter and the non‐planar angle were smaller than those of the AF group (all *p* < 0.05). The RFA group had a greater annulus height in the middle‐systolic phase but a lower annulus height in the end‐systole phase (all *p* < 0.05), and the annular displacement of the RFA group was greater than that of the AF group (*p* = 0.020).

**TABLE 3:** The tricuspid annular parameters derived from 3D echocardiography analysis.

	AF group (*n* = 30)	RFA group (*n* = 30)	Control group (*n* = 30)	*p* ^a^	*p* ^b^	*p* value
**End‐diastolic parameters**						
TA area(cm^2^)	10.85(9.29–12.27)	8.68(7.1–10.46)	7.29(6.19–8.82)	0.009	0.123	<0.001
TA area(cm^2^/m^2^)	6.34(5.46–7.04)	4.88(4.12–6.32)	4.28(3.62–5.16)	0.012	0.207	<0.001
TA circumference(cm/m^2^)	7.31(6.46–7.94)	6.31(5.78–7.01)	5.95(5.44–6.76)	0.009	0.594	<0.001
TA AP diameter(cm/m^2^)	2.26 ± 0.3	1.98 ± 0.32	1.87 ± 0.33	0.003	0.537	<0.001
TA SL diameter(cm/m^2^)	2.08 ± 0.32	1.89 ± 0.31	1.75 ± 0.34	0.062	0.311	0.001
TA sphericity index	1.09 ± 0.10	1.05 ± 0.08	1.08 ± 0.10	0.311	0.987	<0.001
TA non‐planar angle(°)	175.16(157.5–177.55)	162.75(156.76–168.59)	163.53(157.04–168.27)	0.060	1.000	0.015
TA height(cm)	0.84(0.68–0.98)	1.04(0.95–1.2)	0.93(0.82–1.08)	<0.001	0.147	0.001
TL tenting volume(mL)	0.8(0.62–1.3)	0.91(0.67–1.32)	0.84(0.69–0.99)	1.000	1.000	0.749
TL tenting height(cm)	4.93(2.81–8.88)	3.63(2.91–4.64)	2.68(2.02–4.27)	0.267	0.108	0.005
TL tenting area(cm^2^)	0.98(0.59–1.47)	0.74(0.51–1.02)	0.6(0.37–0.84)	0.312	0.303	0.01
**Mid‐systolic parameters**						
TA area(cm^2^)	10.08(8.45–11.85)	8.03(6.11–9.9)	6.02(5.48–7.84)	0.018	0.012	<0.001
TA area(cm^2^/m^2^)	5.94(5.02–6.74)	4.53(3.92–6.09)	3.76(3.29–4.68)	0.015	0.030	<0.001
TA circumference(cm/m^2^)	6.97(6.33–7.66)	6.07(5.54–6.75)	5.55(5.23–6.33)	0.012	0.243	<0.001
TAAP diameter(cm/m^2^)	2.15 ± 0.3	1.91 ± 0.31	1.76 ± 0.32	0.009	0.225	<0.001
TA SL diameter(cm/m^2^)	2.01 ± 0.31	1.81 ± 0.31	1.65 ± 0.3	0.036	0.128	<0.001
TA sphericity index	1.08 ± 0.09	1.06 ± 0.1	1.08 ± 0.11	1.000	1.000	0.792
TA non‐planar angle(°)	170.11(153.1–175.72)	156.41(150.7–165.01)	157.72(149.72–163.23)	0.03	1.000	0.006
TA height(cm)	0.74(0.54–0.89)	0.92(0.76–1.02)	0.79(0.66–0.98)	0.033	0.201	0.026
TL tenting volume(cm^3^)	1.21(1.04–2.23)	1.51(1.16–2.06)	1.4(1.18–1.58)	1.000	0.882	0.534
TL tenting height(cm)	6.97(5.01–11.06)	5.76(4.83–6.9)	5.78(4.5–6.69)	0.132	1.000	0.083
TL tenting area (cm^2^)	1.33(0.53–2.29)	1.15(0.67–1.59)	0.91(0.77–1.03)	0.945	0.234	0.109
TA displacement(mm)	9(5.96–11.1)	12.17(8.2–14.79)	13.26(9.96–15.26)	0.020	1.000	0.001
**End‐systolic parameters**						
TA area(cm^2^)	10.25(8.15–11.67)	7.98(6.01–9.92)	5.79(5.32–7.62)	0.024	0.027	<0.001
TA area(cm^2^/m^2^)	5.75(4.91–6.73)	4.46(3.58–6.05)	3.69(3.13–4.44)	0.021	0.045	<0.001
TA circumference(cm/m^2^)	6.97(6.28–7.65)	5.98(5.49–6.56)	5.46(5.09–6.34)	0.009	0.168	<0.001
TA AP diameter(cm/m^2^)	2.12 ± 0.31	1.89 ± 0.31	1.75 ± 0.31	0.018	0.224	<0.001
TA SL diameter(cm/m^2^)	1.98 ± 0.32	1.78 ± 0.32	1.62 ± 0.28	0.045	0.129	<0.001
TA sphericity index	1.07 ± 0.09	1.07 ± 0.11	1.08 ± 0.10	1.000	1.000	0.838
TA non‐planar angle(°)	167.43(151.89–171.71)	155.76(148.14–162.52)	156.03(148.65–159.33)	0.048	1.000	0.004
TA height(cm)	0.62(0.48–0.83)	0.85(0.66–0.92)	0.71(0.57–0.87)	0.087	0.339	0.054
TL tenting volume(cm^3^)	2.98(1.69–3.55)	2.77(1.99–3.42)	2.22(1.98–2.32)	1.000	0.012	0.016
TL tenting height(cm)	10.19(8.12–14.06)	8.42(7.66–9.25)	8.08(7.35–8.69)	0.009	0.642	0.001
TL tenting area(cm^2^)	2.26(0.91–3.65)	1.81(1.27–2.6)	1.51(0.97–1.53)	0.417	0.03	0.007

*Notes*: Data are presented as mean ± standard deviation, median (Q1–Q3) or percentage. *p* ^a^, RFA group compared with the AF group. *P* ^b^, RFA group compared with the control group.

Abbreviations: AF, atrial fibrillation; AP, anteroposterior diameter; RFA, radiofrequency ablation; SL, septal–lateral diameter; TA, tricuspid annulus; TL, tricuspid leaflets.

Compared with the control group, the RFA group had a larger index area both in middle‐systole and end‐systole (all *p* < 0.05). In the end‐systolic phase, the tricuspid leaflet tenting volume and the tricuspid leaflet tenting area were greater in the RFA group (all *p* < 0.05). There were no significant differences in any of the TA index parameters between the RFA group and the control group in the end‐diastolic phase (all *p* > 0.05). The other parameters did not significantly differ between middle‐systole and end‐systole phases (all *p* > 0.05).

Using stepwise multiple linear regression to identify determinants of TA circumference, six variables were ultimately retained in the model: RFA, age, BSA, DM, TA area, and TA height. The final multivariate model was statistically significant (*F* = 91.407, *p* < 0.001), with 91.2% of the variance in TA reverse remodeling explained by RFA, BSA, younger age, absence of DM, a smaller TA area, and a lower TA height (adjusted *R* ^2^ = 0.912). Standardized regression coefficients (*β*) with corresponding 95% confidence intervals for each independent variable are presented in Table 4.

**TABLE 4:** Multivariate linear regression analysis of the factors associating to the tricuspid annulus circumference.

ANOVA						
Model	Sum of squares	df	Mean square	*F*	*p*	Adjusted *R* ^2^
Regression	77.172	6	12.862	91.407	<0.001	0.912
Residual	7.458	53	0.141			
Total	84.630	59				

					95% CI of B		
Model	*B*	*β*	*t*	*p* value	Lower limit	Upper limit	Collinearity (VIF)
RFA	−0.440	−0.185	−3.767	<0.001	−0.675	−0.206	1.457
Age	−0.011	−0.089	−2.117	0.039	−0.021	−0.001	1.055
BSA	−2.565	−0.448	−10.402	<0.001	−3.060	−2.071	1.116
DM	0.374	0.122	2.795	0.007	0.106	0.643	1.144
TA annulus area‐ED (cm^2^/m^2^)	0.525	0.697	14.953	<0.001	0.454	0.595	1.305
TA annulus height‐ED	0.489	0.111	2.416	0.019	0.083	0.894	1.269

Abbreviations: BSA, body surface area; DM, diabetes mellitus; ED, end‐diastolic phase; RFA, radiofrequency ablation; TA, tricuspid annulus.

### 3.4 Sensitivity Analysis

Three sensitivity analyses were performed to confirm the robustness of the association between RFA and reverse remodeling of the TA circumference in patients with persistent AF (Additional file 1). In Model 1, after additionally adjusting for gender, age, and BSA, RFA was associated with a significant reduction in TA circumference (B = −0.971, 95% CI: −1.432 to −0.510; *p* < 0.001). Model 2 and Model 3 sequentially incorporated additional covariates, including SBP, DBP, hypertension, DM, heart failure, stroke, smoking, alcohol consumption, degree of tricuspid valve regurgitation, medication regimens, and TA area measurements. Both models consistently demonstrated a significant association between RFA and reduced annular circumference.

## 4 Discussion

Using quantitative 3DE with Tomtec analytical software, this study systematically compared TA geometry and right heart RH functional alterations at the 3‐month follow‐up after RFA in persistent AF patients with or without concomitant TR against two control groups: non‐ablated persistent AF patients and sinus rhythm controls. The main findings of this study can be summarized as follows: (1) RFA has the capacity to reverse RA remodeling in persistent AF patients; (2) despite the absence of significant differences in RV FAC measured by conventional 2D echocardiography, 3DE quantification confirmed RFA‐mediated improvement in the RV ejection fraction; and (3) RFA induced reverse remodeling of the TA, which was characterized primarily by a reduction in the annular circumference.

### 4.1 Right Heart Remodeling in AF Patients With RFA

The impact of RFA on RH remodeling in patients with persistent AF encompasses structural and functional alterations in both the RA and the RV. Our findings demonstrated that, compared with those in the AF group, at 3 months post‐RFA, both the maximum and the minimum RA dimensions showed significant reductions regardless of body surface area (BSA) adjustment. However, no statistically significant differences were observed in the RV end‐diastolic volume (EDV) or the end‐systolic volume (ESV).

**FIGURE 3:**
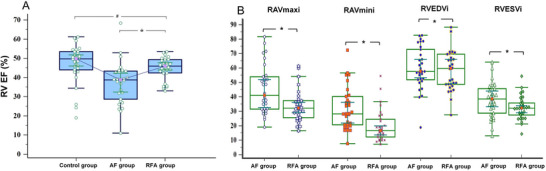
Comparison of RH function among the three groups and structural differences between the AF and RFA groups. (A) Comparison of RH function measured by 3DE among the three groups. RH, right heart; RV EF, right ventricular ejection fraction. (B) Comparison of the geometric structure differences among the AF and RFA groups. AF, atrial fibrillation; RAVmaxi, the maximum diameter of the right atrial volume, adjusted by the BSA; RAVmini, the minimum diameter of the right atrial volume, adjusted by the BSA; RFA, radiofrequency ablation; RV EDVi, the right ventricular end‐diastolic volume, adjusted by the BSA; RV ESVi, the right ventricular end‐systolic volume, adjusted by the BSA. BSA, body surface area. *p < 0.05 for the RFA group compared with the AF group, *^$^p *> 0.05 for the RFA group compared with the AF group, *^#^p *> 0.05 for the RFA group compared with the control group.

In contrast to those in the control group, the RA maximum and minimum dimensions and the RV EDV/ESV remained significantly elevated at 3 months post‐RFA. These results suggested that the initiation of RA reversed remodeling within 3 months post‐ablation. However, the RV EDV and ESV only exhibited decreasing trends without statistically significant differences. Notably, the BSA‐adjusted RV EDV tended to increase, potentially indicating RV functional improvement. Moreover, the RV EF calculated by 3DE showed significant improvement, providing definitive evidence of markedly enhanced RV function at 3 months following RFA. However, no statistically significant differences were observed in the RV FAC assessed by 2DE, suggesting the superior sensitivity of 3DE in detecting post‐RFA changes in RV function among patients with persistent AF (Figure 3).

### 4.2 Tricuspid Annular Remodeling in AF Patients

This study elucidates the multidimensional remodeling effects of RFA on TA geometry in AF patients, encompassing alterations in circumference, annular area, AP diameter, SL diameter, and height. Emerging evidence indicates that AF itself induces right atrial dilation and TA structural remodeling, whereas rhythm control through RFA‐mediated sinus rhythm restoration may reverse these pathological changes [8].

**FIGURE 4:**
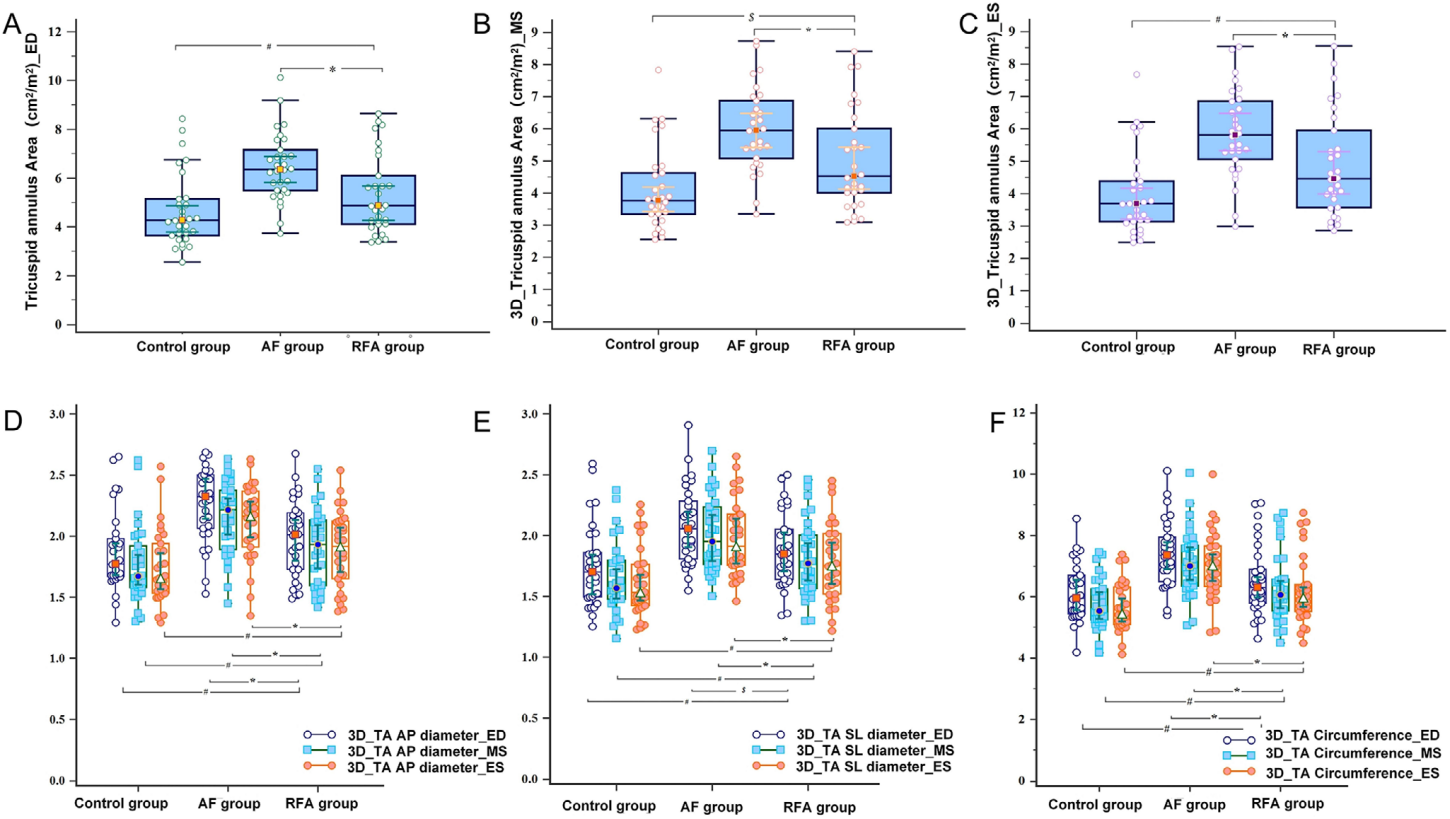
Comparison of the TA parameters among the three groups. (A) Comparison of the 3D TA area in the end‐diastole (ED) phase. (B) Comparison of the 3D TA area in the midsystole (MS) phase. (C) Comparison of the 3D TA area in the end‐systole (ES) phase. (D) Comparison of the 3D TA AP diameter in the ED, MS, and ES phases. (E) Comparison of 3D TA SL diameter in the ED, MS, and ES phases. (F) Comparison of 3D TA circumference in the ED, MS, and ES phases. 3D, three dimensions; AP, anteroposterior diameter; SL, septal–lateral diameter; TA, tricuspid annulus. *p < 0.05 for the RFA group compared with the AF group, *^$^p *< 0.05 for the RFA group compared with the control group, *^#^p *> 0.05 for the RFA group compared with the control group.

Our findings demonstrate three principal observations. First, compared with patients in the AF group, RFA‐treated patients exhibited significant reductions in the TA area during end‐diastole, mid‐systole, and end‐systole, irrespective of BSA adjustment status (Figure 4). Notably, compared with that in the control group, the TA area in the RFA group was not significantly different at end‐diastole, but a larger TA area was observed in the RFA group during both mid‐systolic and end‐systolic phases. This temporal disparity in recovery patterns suggested that following sinus rhythm restoration via RFA in persistent AF patients, early‐phase improvement in TA dimensions predominantly presents during the end‐diastolic phase, whereas systolic‐phase alterations require extended longitudinal monitoring.

Second, the TA circumference significantly decreased across all cardiac phases compared with that in the AF group. However, compared with those in the control group, there were no significant differences in TA circumference in either the diastole phase or the systolic phase (Figure 4), which indicates a full restoration of TA geometry, approximating normal physiological ranges. Thus, the remodeling of the TA circumference may have occurred earlier than the remodeling of the TA area. Therefore, owing to the non‐planar structure of the TA, the non‐planar angle of the TA should be smaller in the RFA group than in the AF group. Our data demonstrated that the TA non‐planar angle was smaller and that the tricuspid leaflet tenting height was significantly lower in the RFA group. Moreover, there were no significant differences in the TA non‐planar angle or tricuspid leaflet tenting height between the RFA group and the control group (Table 3). These findings suggest that RFA may induce reverse remodeling of the non‐planar geometry of the TA.

Furthermore, compared with those in the AF group, quantitative analysis revealed significant reductions in both the tricuspid annular AP diameter and the SL diameter across cardiac cycles in the RFA group. Crucially, these parameters achieved approximately complete anatomical normalization relative to those of the control group, collectively indicating prompt reverse annular remodeling manifesting as early as 3 months post‐RFA procedural follow‐up.

### 4.3 Comparison to Previous Studies on Right Heart and Tricuspid Annular Remodeling

In patients with persistent AF, long‐term irregular atrial contractions lead to RA enlargement, which subsequently induces TA dilation and consequent TR. The progression of TR further exacerbates RA dilation, establishing a vicious cycle [14]. Following RFA, which restores normal sinus rhythm, the resumption of sequential atrial and ventricular contractions may theoretically alleviate TR and prevent further RA dilatation. Notably, in patients with preserved left ventricular function, AF has been shown to induce greater TA dilation than mitral annular dilation does [4, 15]. A previous study using 3.0 T cardiac magnetic resonance (CMR) revealed progressive RA dilation and a significant increase in sphericity in patients with paroxysmal and persistent AF compared with healthy volunteers, but RA fibrosis was similar among the groups [16].

The unique non‐planar saddle‐shaped configuration of the TA [10, 11] poses challenges for comprehensive morphological assessment via conventional 2DE [10, 17]. Therefore, Tomofumi et al. utilized MDCT to evaluate TA remodeling after RFA and demonstrated that patients with AF exhibited early TA dilation and flattening. Rhythm control therapy induced reverse TA remodeling with concomitant reductions in the RAV. Notably, the SL diameter showed more pronounced changes than did the AP diameter during this process [8]. Another study employing MDCT to evaluate RA remodeling in patients with paroxysmal AF following RFA revealed that increased RAV served as an independent predictor of AF recurrence. This finding suggested that persistent structural remodeling of the RA post‐RFA may compromise therapeutic outcomes [18]. Taken together, previous studies have highlighted the importance of RA and TA remodeling in AF patients; however, our study extends these findings by providing detailed 3DE‐based quantification of both structural and functional parameters.

In contrast to 2DE, CT and CMR, 3DE enables complete visualization of the annular architecture through volumetric reconstruction without radiation exposure risk, and it is more accessible in a broader range of regions [12]. Hajo et al. evaluated patients with paroxysmal AF (*n* = 79) and patients with chronic AF (*n* = 19) via 3DE before and after RFA, and a significant reduction in the RAV was observed post‐RFA, regardless of the AF recurrence status, suggesting that RFA may promote reverse RA remodeling. However, the study revealed no significant changes in RV structural or functional parameters at the 6‐month follow‐up after RFA, although improvements in strain parameters were documented [19].

Our study employed Tomtec quantitative analysis software to evaluate TA and RH remodeling in patients with persistent AF. We also found reverse remodeling of both the TA and the RA across multiple parameters post‐RFA, including reductions in annular circumference, area, AP diameter, SL diameter, annular height, and RA dimensions. Interestingly, we found that the RA EF and the RV EF calculated by 3DE were significantly improved, providing definitive evidence of markedly enhanced RA and RV function at 3 months following RFA. However, no statistically significant differences were observed in the RV FAC assessed by 2DE, suggesting that 3DE exhibited superior sensitivity compared with 2DE in assessing right ventricular functional changes. Aleksandra et al. also reported that there was a reduction in the RAV alongside improved strain parameters in both the RV and bilateral atria after RFA, although no significant improvement in the left ventricular ejection fraction (LVEF) was detected. These findings suggest that RFA may have positive effects on the functional profiles of the RA and RV [20].

### 4.4 Limitations and Future Perspectives

This study has several limitations. First, as a single‐center small sample size observational study, potential selection bias may exist. Second, the AF group had a more proportion of patients with moderate‐to‐severe (≥2+) TR than did the RFA group. Given the bidirectional relationship between TR and remodeling of both the TA and the RA [21], this confounding factor might obscure the isolated effects of RFA on these structural changes. However, this observation implies that RFA may ameliorate TR through reverse remodeling of TA and RH structures. Finally, the cross‐sectional design of this study necessitates further validation through longitudinal cohort studies to characterize the long‐term impact of RFA on TA and RH remodeling patterns.

## 5 Conclusions

Using 3DE and Tomtec quantitative analysis software, our results demonstrated that RFA contributes to improving TA and RH reverse remodeling in patients with persistent AF. Moreover, 3DE could offer more comprehensive and sensitive evaluations with excellent feasibility, facilitating readily accessible evaluations for AF patients undergoing RFA. Further studies are needed to observe the long‐term impact of RFA on TA and RH reverse remodeling.

## Funding

This study was supported by grants from the Science and Technology Planning Project of Xiamen (grant number: 3502Z20214ZD1166), the Science and Technology Projects in Guangzhou (grant number: 2023A04J2276), and the National Foreign Expert Program (grant number: G2023030055L).

## Ethics Statement

The study was approved by the Ethics Committee of Xiamen Cardiovascular Hospital (No. XXY‐AF/SC‐09.01/2.0).

## Consent

All participants provided written informed consent.

## Conflicts of Interest

The authors have no competing interests to declare.

## Supporting information



Additional file 1. Multiple Linear Regression: RFA and TA Circumference Reverse Remodeling (Sensitivity Analysis)

## Data Availability

All data generated or analyzed during this study are included in this published article and its supplementary information files.
