# Supplementary material for: Evaluation of the Remodeling of the Tricuspid Annulus and Right Heart in Persistent Atrial Fibrillation Patients With or Without Radiofrequency Ablation via Three‐Dimensional Echocardiography
Source: Echocardiography. 2025 Oct 22;42(10):e70319. doi: 10.1111/echo.70319 (PMC12542883; doi:10.1111/echo.70319)
Supplement: Supplementary file 1 — Additional file 1. Multiple Linear Regression: RFA and TA Circumference Reverse Remodeling (Sensitivity Analysis) [file ECHO-42-e70319-s001.docx]

**Additional file 1. Multiple Linear Regression: RFA and TA Circumference Reverse Remodeling (Sensitivity Analysis)**

| RFA | Model 1 | | Model 2 | | Model 3 | |
| --- | --- | --- | --- | --- | --- | --- |
|  | B （95% CI） | p | B （95% CI） | p | B （95% CI） | p |
|  | -0.971 (-1.432, -0.510) | <0.001 | -0.832 (-1.346, -0.318) | 0.002 | -0.374 (-0.650, -0.097) | 0.009 |

RFA, Radiofrequency Ablation; TA, tricuspid annulus.

Model 1: adjusted by gender, age, body surface area

Model 2: adjusted by gender, age, body surface area, systolic blood pressure, diastolic blood pressure, hypertention, diabetes mellitus, heart failure, stroke, smoking, tricuspid regurgitation severity.

Model 3: adjusted by gender, age, body surface area, systolic blood pressure, diastolic blood pressure, hypertention, diabetes mellitus, heart failure, stroke, smoking, tricuspid regurgitation severity, angiotensin II receptor blocker, angiotensin receptor-neprilysin inhibitor, beta-blocker, sodium-glucose cotransporter 2 inhibitor, tricuspid annulus area in end-diastole phase.
